# Raman-dielectrophoresis goes viral: towards a rapid and label-free platform for plant virus characterization

**DOI:** 10.3389/fmicb.2023.1292461

**Published:** 2023-11-22

**Authors:** Alessio Sacco, Giulia Barzan, Slavica Matić, Andrea M. Giovannozzi, Andrea M. Rossi, Chiara D’Errico, Marta Vallino, Marina Ciuffo, Emanuela Noris, Chiara Portesi

**Affiliations:** ^1^Quantum Metrology and Nano Technologies Division, National Institute of Metrological Research (INRiM), Torino, Italy; ^2^Institute for Sustainable Plant Protection, National Research Council of Italy (CNR), Torino, Italy

**Keywords:** Raman spectroscopy, dielectrophoresis, plant viruses, TMV, virus-derived nanoparticles

## Abstract

An innovative spectroscopic method that allows to chemically and structurally characterize viruses directly in suspension within few minutes was developed. A library of five different plant viruses was obtained combining dielectrophoresis (DEP), performed with a device specifically designed to capture and agglomerate virus particles, and Raman spectroscopy to provide a chemical fingerprint of virions. The tested viruses, purified from infected plants, were chosen for their economic impact on horticultural crops and for their different morphological and structural features. Using the Raman-DEP device, specific profiles for each virus were successfully obtained, relying on chemical differences occurring even with genetically similar viruses belonging to the same taxonomic species and morphologically indiscernible by transmission electron microscopy (TEM). Moreover, we investigated the potentiality of Raman-DEP to follow dynamic changes occurring upon heat treatment of tobacco mosaic virus (TMV) particles. Raman peak deviations linked to TMV coat protein conformation were observed upon treatment at temperatures equal or higher than 85°C, substantiating the rod-to-spherical shape transitions observed by TEM and the concomitant drastic loss of infectivity following plant inoculation. Overall, the Raman-DEP method can be useful for the characterization of virus (nano)particles, setting the basis to create a database suitable for the study of viruses or virus derived-nanoparticles relevant for the agricultural, medical, or biotechnological fields.

## Introduction

Recently, a dramatic increase in viral outbreaks is occurring worldwide, provoking severe consequences on human, animal, and plant health (Baker et al., 2022). Despite the reduced perception of the economic and social impact of viral diseases in agriculture compared to epidemics occurring in humans and animals, plant viruses cause economic losses, accounting to up to 50 billion €/year worldwide (Pallás et al., 2018), changing the agricultural landscape and increasing food insecurity (Rybicki, 2015). Considering the larger diffusion of viruses due to the ongoing climate change situation and the greater demand for food availability (Hilaire et al., 2022), it is of great interest to develop innovative techniques to diagnose and characterize plant viruses (Cassedy et al., 2020), using strategies alternative to traditional methods.

Raman spectroscopy (RS) relies on the phenomenon of inelastic light scattering by the molecules present in a sample after their excitation to higher rotational or vibrational slates. RS offers interesting advantages in the analytical sector, thanks to its versatility, specificity, rapidity, and simplicity in sample preparation, allowing to perform dynamic analysis and providing structural and chemical information at the molecular level (Tuma, 2005), with an eco-friendly approach (Barzan et al., 2021). RS is a non-destructive technique, enabling to directly detect the chemical modifications occurring in a matrix, e.g., a plant leaf, during pathogen infection, even in the absence of visible symptoms (Mandrile et al., 2019, 2022). In this respect, RS can be applied directly as a “point-of-care” test (Peeling et al., 2006) even by untrained users (Chen et al., 2019; Vidic et al., 2019), at low operating costs.

Moreover, RS-based approaches have been used to characterize directly viruses (Němeček and Thomas, 2009) and plant viruses, such as turnip yellow mosaic virus (Hartman et al., 1978), bean pod mottle virus (Li et al., 1990, 1993), belladonna mottle virus (Prescott et al., 1985), bacteriophages (Fish et al., 1980; Aubrey et al., 1992; Overman and Thomas, 1995; Tuma et al., 1996); more recently RS has been used to deeply investigate an echovirus (Ruokola et al., 2014) and an algal infecting coccolithovirus (Yakubovskaya et al., 2021). Since RS is not affected by the presence of water in the sample, it is possible to perform direct analysis of isolated or purified small biological particles, such as viruses suspended in aqueous environments. However, viruses are rarely analyzed in suspension due to their low Raman cross-section, thus hindering the efficiency of this approach. To overcome this limitation and to enhance the Raman signal of viral particles, in this work the forces generated by dielectrophoresis (DEP) via non-uniform electric fields in liquid were exploited and a DEP device was combined with RS. Indeed, DEP can force the agglomeration of suspended particles, such as biomolecules, nanoparticles, bacteria, or other microorganisms (Castellarnau et al., 2006; Khoshmanesh et al., 2011).

The combination of Raman and DEP was already successfully applied for the rapid characterization of bacteria and the detection of antibiotic resistance, in alternative to classical microbiological techniques (Barzan et al., 2020). Raman-DEP allowed also to study dynamic interactions of bacteria with different biocides, monitoring in real-time the spectral profiles of the microbes (Barzan et al., 2022).

Here, a new DEP cell was developed for the analysis of viruses, and the Raman-DEP approach was applied for the first time to characterize plant-infecting viruses, selected as safer candidates compared to animal-infecting ones. To set up the procedure and test its performances, five different viruses were used, i.e., tobacco mosaic virus (TMV), tomato mosaic virus (ToMV), tomato brown rugose fruit virus (TBFRV), cucumber mosaic virus (CMV), and tomato spotted wilt virus (TSWV). These viruses were selected not only for their worldwide economic importance (Scholthof et al., 2011), but also for their characteristic and distinct particle morphology and genomic elements, determining their assignment to different taxonomic families.

TMV, ToMV, and TBRFV belong to the genus *Tobamovirus*, the largest taxonomic group within the *Virgaviridae* family, and are characterized by rigid virions with a predominant length of 300–310 nm and a diameter of 18 nm. Tobamoviruses possess a non-segmented positive linear single-stranded RNA (ssRNA) genome, encoding three non-structural proteins which are required for viral replication and movement, and a structural capsid protein (CP) (17–18 kDa). TMV is the most well-characterized virus from the structural point of view; its virions consist of 2,130 identical copies of the monomeric CP, assembled in a helical arrangement. In nature, tobamoviruses are transmitted mechanically during agricultural practices or through seeds.

CMV (genus *Cucumovirus,* family *Bromoviridae*) is a multicomponent virus whose genome consists of three ssRNA molecules individually encapsidated in isometric icosahedral particles of 28 nm, made of a single CP (Palukaitis et al., 1992). Beside CP, the CMV genome encodes four non-structural proteins. CMV has the widest host range and in nature can be transmitted by aphids in a stylet-borne, non-persistent manner (Nault, 1997).

TSWV (genus *Orthotospovirus* family *Tospoviridae*) has an extremely wide host range, infecting up to 1,100 different crop and weed species. Virions appear as spherical particles with a diameter of 80–120 nm, surrounded by a membrane. Two glycoproteins (G1 and G2) protrude from the virion surface and govern the transmission by thrip vectors. The TSWV genome consists of three RNA segments (Turina et al., 2016), each enveloped by several copies of the nucleocapsid (N) protein (Adkins, 2000).

In this work, virions purified from systemically infected plants, whose purity and morphology were checked by transmission electron microscopy (TEM) and sodium dodecyl sulphate-polyacrylamide gel electrophoresis (SDS-PAGE) were used. Following Raman-DEP analysis, tentative assignment of the chemical meaning of each spectral signal was obtained, revealing a very high spectral specificity.

In addition, the potential of Raman-DEP was tested to monitor dynamic changes occurring in TMV particles subjected to different temperature treatments. TMV virions can withstand extremely high temperatures (Zaitlin and Israel, 1975) and, upon heating above 90°C, undergo dramatic particle remodeling, shifting from the typical rod-shaped structure to spherical nanoparticles of 100–800 nm, devoid of RNA (Atabekov et al., 2011; Bruckman et al., 2015). Due to the importance of bioengineering platforms based on viruses (Nkanga and Steinmetz, 2021), particularly of TMV-based nanoparticles, for biotechnological and nanomedicine applications (Alonso et al., 2013; Wege and Koch, 2020), we considered that obtaining RS signatures of TMV virions in their different conformations could be of interest for a broad community.

Overall, the method described in this work can be useful for a direct characterization of viruses, including animal and human pathogens. DEP has already been applied to detect and discriminate viruses, but in most cases it was applied to indirectly measure the different DEP responses of virus-infected and non-infected cells grown in culture (Rahman et al., 2017). Here, the analysis was conducted directly on virion particles, allowing for the first time a precise chemical characterization of viral particles in liquid medium.

## Materials and methods

Further details on the methods used in this work are presented in the supplementary material.

### Plants and virus materials

Plants of *Nicotiana benthamiana, N. tabacum*, and *N. clevelandii* were maintained in a greenhouse at 23°C (14/10 h light/dark). The list of isolates of TMV, ToMV, TBRFV, CMV, and TSWV used in this study is presented in Supplementary Table S1. All isolates are from the IPSP-CNR PLAVIT collection and are available through the EVA-GLOBAL platform.^1^

### Virus production and purification

Each virus was mechanically inoculated onto plants using silicon carbide as abrasive and symptomatic leaves were used for virus purification. For the tobamoviruses TMV, ToMV, and TBRFV propagated onto *N. benthamiana*, the purification method of Chapman was essentially followed (Chapman, 1998), while the purification protocols for TSWV and CMV were specifically optimized, as detailed in the Supplementary material.

### Transmission electron microscopy (TEM) examination

Virions were analyzed by negative staining with 0.5% aqueous uranyl acetate. Appropriate dilutions of purified virions were adsorbed onto Pelco^®^ formvar and carbon-coated grids for 5 min. Grids were photographed using a CM 10 electron microscope (Philips, Eindhoven, Netherlands) operating at 60 kV. Micrograph films were developed and digitally acquired at high resolution with a D800 Nikon camera. Images were trimmed and adjusted for brightness and contrast using Fiji software.

### Protein analysis

The protein composition of purified virion preparations was analyzed by SDS-PAGE. Each virion suspension was dissolved in 2× SDS-PAGE gel loading buffer and incubated at 95°C for 3 min. Clarified protein extracts were separated on Mini-Protean TGX acrylamide gradient (4–20%) gels (Bio-Rad, Richmond, CA) in Tris-glycine buffer and gels stained with Coomassie brilliant blue, according to standard procedures.

### Thermal stability of TMV particles

The thermal stability of purified TMV particles was assessed on 50-μl aliquots of purified TMV suspension (7.5 mg/mL). Following incubation in ice for 10 min, virion aliquots were moved separately to thermal blocks set at either 75°C, 85°C, or 90°C for 3 min, or left in ice as control. Afterwards, each treated sample was cooled back in ice for 10 min and then either loaded onto EM grids for TEM observation, used for infectivity assays or for Raman analysis.

### Infectivity assay

Purified TMV virion suspensions (7.5 mg/mL) subjected to different thermal treatments (see above) were mechanically inoculated at 10^−2^, 10^−3^, and 10^−4^ dilutions with silicon carbide onto *N. clevelandii* plants (using three biological replicates, three leaves each). At 5 days post-inoculation, the number of chlorotic lesions developed on leaves was counted.

### Raman-dielectrophoretic (Raman-DEP) analysis

A custom-made DEP sample holder was manufactured as previously detailed (Barzan et al., 2020) and adapted for the analysis of viruses, as detailed in Supplementary Figure S1. A non-uniform electric field was generated by applying alternated voltage to the electrodes at the bottom of the cell (5 V peak-to-peak, 15 MHz frequency), forcing the agglomeration of virions.

For all experiments, aliquots of 5 μL of each purified virus, resuspended in phosphate buffer saline solution (PBS, PanReac Applichem, pH 7.4) were injected into the Raman-DEP device and analyzed with a Thermo Scientific™ DXRxi dispersive Raman microscope, equipped with a 532 nm Nd:YAG excitation laser and an Andor EMCCD spectrograph with a 900 lines/mm grating (spectral range 100–3,300 cm^−1^, spectral resolution 5 cm^−1^). A 60× water immersion, cover-glass corrected objective with 1.1 NA (Olympus LUMFLN60XW) and 1.5 mm working distance was employed in a 180° backscattering collection geometry; transmission white-lamp illumination for bright-field microscopy was used for positioning and for general control of the system.

For each virus, at least 10 acquisitions were taken and averaged; each measurement was performed either in different areas of the DEP cell in which the particles are concentrated, or in sequential measurements, each followed by thorough cell rinsing with purified water. Raman spectra were acquired with a 60 s total integration time (1 s × 60 scans), using an excitation power of 20 mW (measured at the sample). Wavelength and relative intensity were calibrated before each experiment using a neon low-pressure emission lamp and NIST SRM 2242a (Choquette et al., 2007).

## Results and discussion

### Sample purity for Raman-DEP investigation

**Figure 1:**
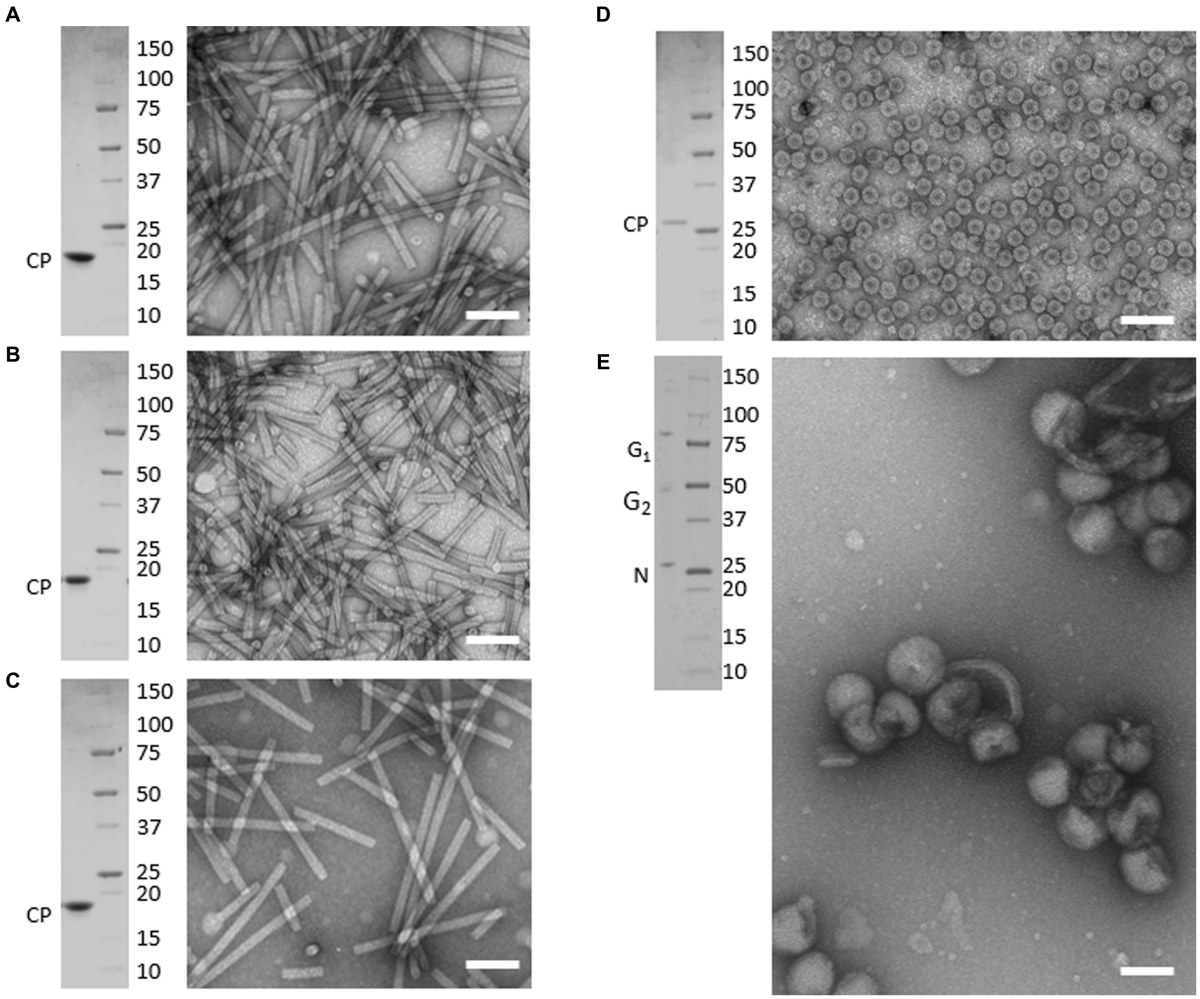
Purified preparations of plant pathogenic viruses used for Raman-DEP characterization. Purified virions were analyzed by transmission electron microscopy (TEM) and denaturing electrophoretic analysis (SDS-PAGE). Each panel includes TEM micrographs showing homogenous populations of intact virions (right) and the Coomassie-stained SDS-PAGE analysis (left), showing that only known structural proteins are associated to each virion preparations, with no contaminants in the range of 250 to 10 kDa. Scale bar, 100 nm. **(A)** TMV; **(B)** ToMV; **(C)** TBRFV; **(D)** CMV; **(E)** TSWV. Molecular mass marker (Precision Plus ProteinTM Standards, BioRad, Richmond, CA) is run as protein mass control.

TEM analysis of virion preparations showed that each sample consisted of highly homogenous populations of intact particles. As shown in Figure 1, tobamovirus virions (ToMV, TMV, and TBRFV) consisted of non-enveloped, rigid, rod-shaped particles of 300 nm × 18 nm, while CMV virions consisted of non-enveloped, icosahedral particles with a 26–35 nm diameter. Finally, TSWV virions appeared as enveloped pleomorphic spherical particles of 80–120 nm, as expected.

When separated in denaturing gel and stained with Coomassie, protein extracts obtained from each virion sample appeared to consist only of the structural proteins characteristic of each virus, with no detectable contaminants in the range from 250,000–10 kDa. Specifically, the tobamovirus CPs migrated next to the 20 kDa marker, according to their calculated molecular mass (MM), i.e., 17.7 kDa for ToMV, 17.6 kDa for TMV, and 17.5 kDa for TBRFV (Figures 1A–C; Supplementary Table S2). The CMV CP migrated slightly higher than the 25 kDa marker, in agreement with its expected MM (24.1 kDa) (Figure 1D; Supplementary Table S2). Finally, in TSWV virion sample, proteins with apparent MM of 28, 55, and 80 kDa were detected (Figure 1E), corresponding to the calculated MM of the nucleoprotein N (28.9 kDa) and of the two virion-associated glycoproteins G1 and G2 (78 and 54 kDa, respectively) (Supplementary Table S2).

### Raman-DEP characterization of individual plant viruses

In the electric conditions applied, considering an estimated medium conductivity of approximately 1.6 S m^−1^, negative DEP was achieved for all virion preparations. After sample injection, the electric field was generated until the confocal volume of the microscope was filled, resulting in a stabilized signal-to-noise ratio of the Raman spectra. The agglomeration time varied according to each virus, i.e., 1 min for TSWV, 3 min for TMV and TBRFV, and 5 min for ToMV and CMV.

**Figure 2:**
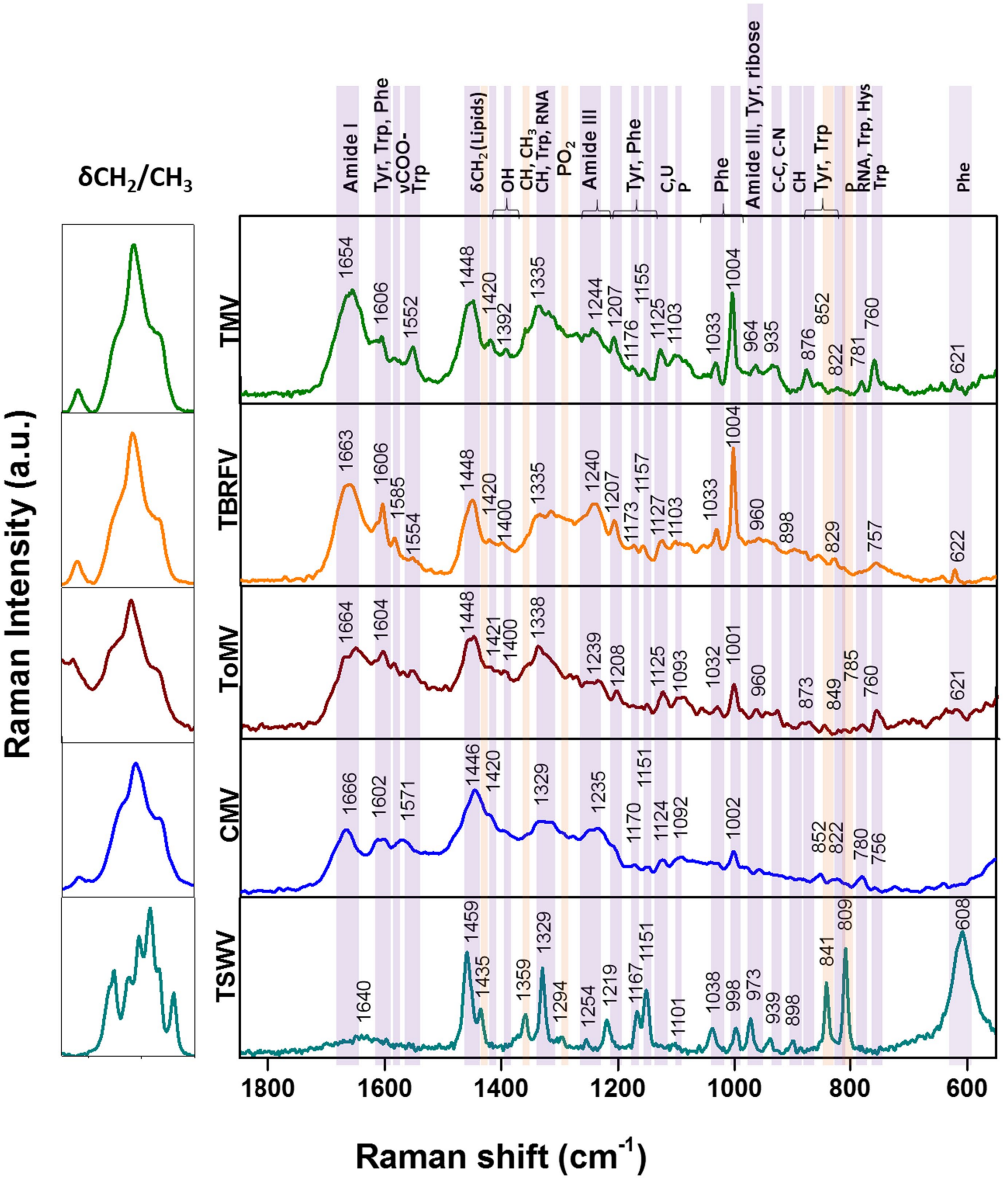
Raman-DEP spectra of aqueous suspension of TMV, TBRFV, ToMV, CMV, TSWV and principal signal assignment. All spectra were normalized for their CH Raman signal (3,000–2,800 cm^−1^) in order to equalize the biological content of each sample. Average spectra of 10 repetitions for each virion preparations are shown.

**Figure 3:**
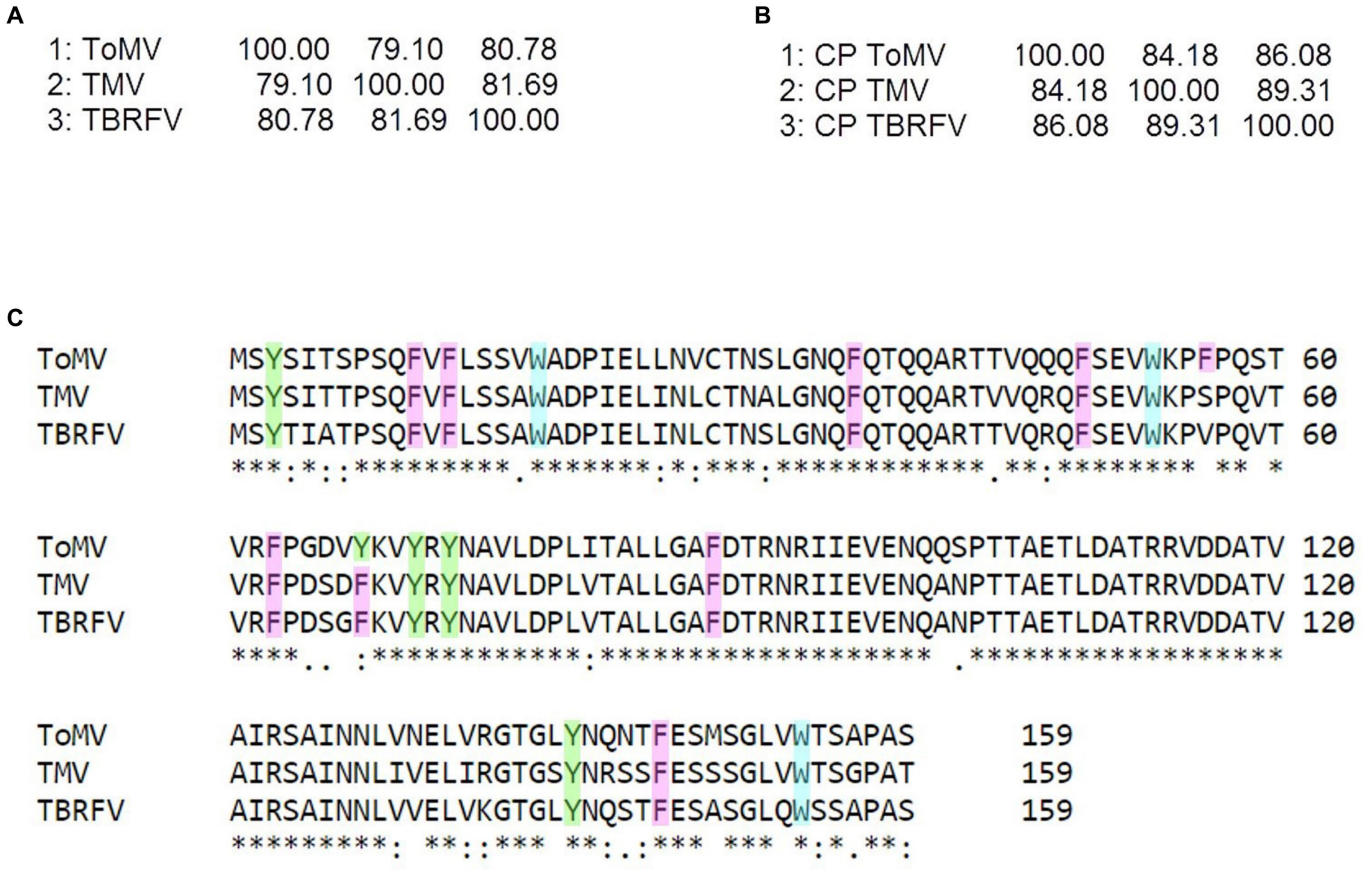
Sequence analyzes of the tobamoviruses TMV, ToMV, and TBRFV. Matrix identity scores of **(A)** whole genome sequences and **(B)** amino acid sequences of the coat proteins (CP) obtained by Clustal Omega (https://www.ebi.ac.uk/Tools/msa/clustalo/). **(C)** Sequence alignment of the CPs of the tobamoviruses with aromatic residues highlighted by different colors (Y, green; F, pink; W turquoise). In the alignment, + denotes amino acid identity among the sequences indicates two identical amino acids, and “no symbol” identifies no identity.

Raman specific signals obtained with the Raman-DEP device from the plant viruses in the regions of the chemical fingerprint between 500 and 3,100 cm^−1^ were considered, and a chemical assignment was made for each signal (Figure 2; Table 1). As shown in Figure 2, non-enveloped viruses show a similar Raman profile in the molecular fingerprint region, likely due to the same basic structural components (proteins and nucleic acids). Their profiles clearly differ from that of TSWV, the only enveloped virus here considered, having a lipid shell decorated by glycosylated proteins. Nonetheless, a more accurate analysis of the spectra of non-enveloped viruses indicated that the profiles of the three tobamoviruses were clearly the most similar. Interestingly, while the Raman spectra of TMV and TBRFV appeared almost indistinguishable, some differences could be discerned compared to ToMV, mainly regarding the amide peaks (amide I at 1654–1664 cm^−1^ and amide III at 960–964 cm^−1^) and the phenylalanine and other aromatic amino acids vibrations (1,606 cm^−1^, 1,200–1,150 cm^−1^; 1,004 cm^−1^; 870–820 cm^−1^; 760 cm^−1^; 620 cm^−1^). Specifically, a different Raman intensity ratio could be observed between bands related to amide I (1654–1,664 cm^−1^) and to aromatic amino acids (Tyr, Trp, Phe at 1606–1604 cm^−1^); furthermore, the peaks at 1004 cm^−1^ and 621 cm^−1^, typical of phenylalanine, appeared narrower and more intense in the TMV and TBFRV spectra compared to ToMV. Moreover, the band at 960–964 cm^−1^, attributable to amide III, tyrosine and ribose, was very pronounced in the TMV and ToMV spectra, but only barely visible for TBRFV. These features, obtained from viruses which are morphologically and structurally very similar (Figure 1) and share high genome identity (79.1–81.69%) (Figure 3A) suggest that RS can sense different biochemical signals, characteristic for each virus.

**Table 1:** Tentative assignment of Raman signals of purified plant viruses.

Assignment	TMV	TBRFV	ToMV	CMV	TSWV	Reference
Amide I	1,654	1,663	1,664	1,666	1,640	1,669 (Turano et al., 1976); 1,660 (Deng et al., 1993)
Tyr, Trp, Phe	1,606	1,606	1,604	1,602	–	1,604–1,607 (Cialla et al., 2009)
νCOO-	–	1,585	–	–	–	1,610–1,550 (Hobson, 2012)
Trp	1,552	1,554	–	1,571	-	1,553–1,559 (Turano et al., 1976)
δCH_2_	1,448	1,448	1,448	1,446	1,459	1,458 (Turano et al., 1976)
COOH	1,420	1,420	1,421	1,420	1,435	1,412–1,416 (Deng et al., 1993)
δOH; COO-	1,392	1,400	1,400	–	–	1,400–1,200 (Hobson, 2012); 1,410 (Hobson, 2012)
δCH; ρCH_3_.	–	–	–	–	1,359	1,340–1,315 (Hobson, 2012); 1,380 (Hobson, 2012)
δCH, Trp, A, G	1,335	1,335	1,338	1,329	1,329	1,333–1,331–1,337-1338-1339 (Turano et al., 1976; Cialla et al., 2009)
PO_2_	–	–	–	–	1,294	1,260–1,200 (Hobson, 2012)
C, A, U, Amide III	–	–	–	–	1,254	1,296 (Turano et al., 1976; Deng et al., 1993)
Amide III	1,244	1,240	1,239	1,235	–	1,230–1,300 (Turano et al., 1976; Ruokola et al., 2014)
Tyr, Phe	1,207	1,207	1,308		1,219	1,214–1,208 (Turano et al., 1976; Ruokola et al., 2014)
Tyr, Phe, CH3	1,176	1,173	–	1,170	1,167	1,178(Turano et al., 1976); 1,156–1,176 (Reilly and Thomas, 1994)
C-N, Tyr	1,155	1,157	–	1,151	1,151	1,159 (Turano et al., 1976); 1,158 (Deng et al., 1993)
C-N, C, U	1,125	1,127	1,125	1,124	–	1,127 (Turano et al., 1976); 1,125 (Deng et al., 1993)
C-N, P	1,103	1,103	1,093	1,092	1,101	1,100 (Turano et al., 1976); 1,091 (Ruokola et al., 2014)
Phe	1,033	1,033	1,032	-	1,038	1,031 (Reilly and Thomas, 1994); 1,045 (Turano et al., 1976)
Phe	1,004	1,004	1,001	1,002	998	1,000–1,005 (Turano et al., 1976), 1,003 (Ruokola et al., 2014)
Amide III, Tyr, ribose	964	960	960	–	973	960–978 (Ruokola et al., 2014); 971 (Cialla et al., 2009); 982 (Deng et al., 1993)
C-C, C-N	935	–	–	–	939	938 (Turano et al., 1976); 931 (Cialla et al., 2009); 932 (Deng et al., 1993)
δCH	–	898	–	–	898	900–880; 890–870 (Hobson, 2012)
Trp, Tyr	876	–	873	–	–	879 (Turano et al., 1976); 872 (Deng et al., 1993); 873 (Ruokola et al., 2014)
Tyr	852–822	829	849	852–822	841	856 (Turano et al., 1976); 825–855 (Ruokola et al., 2014)
P	–	–	–	–	809	812 (Turano et al., 1976); 811 (Ruokola et al., 2014)
C, U, T, Trp, His, Thr	781	–	785	780	–	785 (Turano et al., 1976); 771 (Cialla et al., 2009); 770 (Deng et al., 1993); 783 (Ruokola et al., 2014)
Trp	760	757	760	756	–	759 (Turano et al., 1976); 753 (Cialla et al., 2009); 758 (Ruokola et al., 2014)
Phe	621	622	621	–	608	610 (Deng et al., 1993)

As it can be seen in the matrix identity scores of the structural proteins (CPs) of the three tobamoviruses here considered (Figure 3B), the amino acid sequences of the CPs of TMV and TBRFV share the highest identity score of 89.31%, while ToMV has identity scores of 83.65 and 86.16% with TMV and TBRFV, respectively. Moreover, the CP of ToMV has 16 aromatic residues, i.e., one more compared to TMV and TBRFV (Figure 3C). We can envisage that these differences support the discrepancies observed in the RS profiles.

Plant RNA viruses have the tendency to mutate during vector transmission or host infection, with relevant consequences from the adaptation and evolutionary point of view (Butković and Gonzalez, 2022). To evaluate the level of specificity of the Raman-DEP approach to discriminate tobamoviruses, we examined the level of conservation of the CP amino acid sequences of the three species here compared. Following CP sequence alignment of thirty randomly selected isolates of either TMV, ToMV, or TBRFV, all retrieved from the NCBI Genbank database, a high level of conservation was recorded, including presence and position of the aromatic amino acids whose chemical bonds are detected by RS (Supplementary Figure S2). Whether these few differences encountered in few isolates generate different and specific Raman fingerprints remains to be elucidated.

Considering the spectral profile of CMV, the most evident differences compared to the three tobamoviruses account for a general reduction of the relative intensities of the RS signals. In particular, the δCH2 band appears higher and broader in CMV than in the other viruses. Furthermore, the phenylalanine peaks at 1033 cm^-1^ and 1,002 cm^-1^ (Figure 2; Table 1) are less intense compared to TMV and TBRFV. These features could be supported by the relatively lower percentage of aromatic (6.9%) and phenylamine residues (2.3%) in the CP of CMV compared to the other viruses (Table 2).

**Table 2:** Amino acid composition of the virion-associated proteins.

Amino acid (aa)	ToMV	%	TMV	%	TBRFV	%	CMV	%	TSWV N	%	TSWV G1-G2	%
Ala	12	7.5	14	8.8	16	10.1	19	8.7	19	7.4	46	4.1
Arg	9	5.7	11	6.9	9	5.7	21	9.6	8	3.1	32	2.8
Asn	11	6.9	10	6.3	10	6.3	7	3.2	11	4.3	63	5.6
Asp	7	4.4	8	5.0	7	4.4	15	6.9	15	5.8	62	5.5
Cys	1	0.6	1	0.6	1	0.6	1	0.5	3	1.2	46	4.1
Gln	12	7.5	9	5.7	11	6.9	6	2.8	9	3.5	28	2.5
Glu	7	4.4	7	4.4	7	4.4	6	2.8	17	6.6	64	5.6
Gly	6	3.8	6	3.8	6	3.8	11	5.0	12	4.7	61	5.4
His	0	0.0	0	0.0	0	0.0	3	1.4	2	0.8	19	1.7
Ile	7	4.4	9	5.7	7	4.4	9	4.1	19	7.4	108	9.5
Leu	13	8.2	12	7.5	13	8.2	20	9.2	28	10.9	97	8.5
Lys	2	1.3	2	1.3	3	1.9	12	5.5	31	12.0	83	7.3
Met	2	1.3	1	0.6	1	0.6	4	1.8	9	3.5	19	1.7
Phe	8	5.0	8	5.0	8	5.0	5	2.3	12	4.7	56	4.9
Pro	8	5.0	8	5.0	8	5.0	14	6.4	5	1.9	47	4.1
Ser	15	9.4	16	10.1	14	8.8	24	11.0	22	8.5	109	9.6
Thr	16	10.1	16	10.1	16	10.1	12	5.5	14	5.4	80	7.0
Trp	3	1.9	3	1.9	3	1.9	1	0.5	0	0.0	11	1.0
Tyr	5	3.1	4	2.5	4	2.5	9	4.1	7	2.7	48	4.2
Val	15	9.4	14	8.8	15	9.4	19	8.7	15	5.8	56	4.9
Aromatic aa	16	10.06	15	9.43	15	9.43	15	6.88	19	7.4	147	12.95

TSWV, the only virus tested surrounded by a lipidic shell on its surface showed a very different Raman profile. Compared to the non-enveloped viruses here considered, most of the differences rely on the CH bonds vibrational regions (3000–2,800 cm^-1^, 1,459 cm^-1^, 1,329 cm^-1^) which are the most representatives of lipids, whose peaks appear narrower and higher for TSWV.

The intensity of the bands at 1445 cm-1 and 1,660 cm-1 deriving from the C-H and the amide I vibrational modes, respectively, can be useful to calculate the lipid/protein ratio, a valuable feature to discriminate the structural composition of the samples analyzed.

However, other spectral discrepancies allow to distinguish TSWV from non-enveloped viruses, such as (i) the C-O and C-N bonds signals, absent in the TSWV spectrum and (ii) a very sharp and intense peak due to P-O vibrations at 809 cm-1, present only in TSWV. This peak suggests a possible phosphorylation of the TSWV virion-associated proteins. Following a predictive search for phosphorylation sites of the TSWV N protein using NetPhos – 3.1,^2^ up to 30 putative phosphorylation sites at serine, threonine, and tyrosine residues were identified (Supplementary Figure S3). Indeed, phosphorylation of the structural proteins N and CP of RNA viruses can influence the infection process and the assembly and stability of virions, modulating the interaction between structural proteins and nucleotides. Currently, it is unknown if the virion-associated TSWV N protein is phosphorylated *in vivo*, but it is interesting to note that phosphorylation of the N protein of groundnut bud necrosis virus, a TSWV-related orthotospovirus, can occur *in vitro* thanks to the intervention on plant-associated kinases (Bhat and Savithri, 2011). Accordingly, N protein phosphorylation occurs for animal-infecting bunyaviruses, the taxonomic group that includes the plant-infecting orthotospovirus (Yoon et al., 2015).

Furthermore, Raman signals related to phenylalanine and other aromatic residues were reduced or even absent in the TSWV spectrum compared to non-enveloped viruses; this could result from a partial masking by the prominent CH signals relevant to the higher lipids/proteins ratio. Considering the number of aromatic residues in the structural proteins of the viruses tested, we observed that the TSWV N and G1-G2 proteins have 7.4% and up to 13% aromatic residues, respectively (Table 2), supporting the possibility that these peaks are hidden by those related to other chemical bonds.

### Raman-DEP detects structural changes in heat-treated TMV virions

TMV particles maintained at different temperatures were subjected to Raman-DEP analysis, to ascertain whether differences linked to chemical and/or structural alterations occurring in the transition from intact to disrupted virions could be ascertained. For this, the same batch of purified TMV virions used above was incubated at temperatures ranging from 75°C to 90°C, based on published reports (Atabekov et al., 2011).

**Figure 4:**
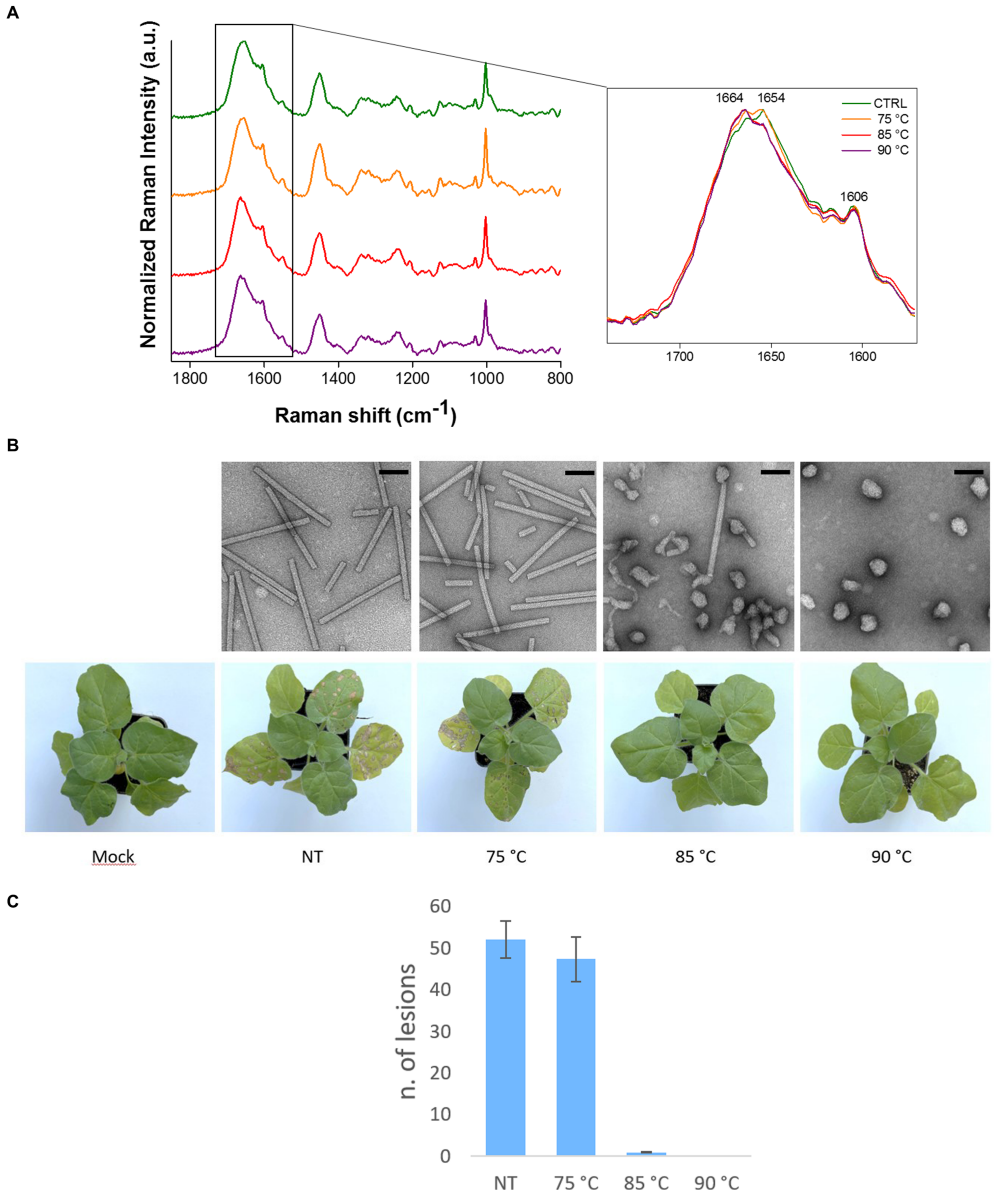
Raman-DEP analysis of heat treated TMV virions. **(A)** Raman-DEP spectra of aqueous suspension of TMV virions subjected to different heat treatments. Average spectra of 10 repetitions for each virion preparations are shown. A detail of the peaks in the 1,580–1740 cm-1 range of the spectra of virions left untreated (CTRL) or after heat treatment at the indicated temperatures is shown enlarged. [**(B)**, upper part] TEM micrographs of negatively-stained TMV virions left untreated (NT) or subjected to the indicated temperatures. Magnification bar: 100 nm. [**(B)**, lower part] Residual infectivity (local lesions) of TMV particles kept in ice (Not-Treated, NT) or subjected to heat treatment. A plant inoculated with the inoculation buffer alone is shown as control (Mock). The TMV inoculum consisted of purified virions (75 μg/mL). **(C)** Number of local lesions developed on leaves inoculated with TMV virions left untreated (NT) or treated at the indicated temperatures. Error bars represent standard errors of the mean.

As shown in Figure 4A, the Raman spectra of heat-treated TMV particles deviate from that of untreated control samples in the region including the peaks related to amide I (1660–1,680 cm^-1^). This was particularly evident for virions subjected to temperatures ≥85°C. A specific change occurs in a spectral region known to be related to the O=C–NH peptide bond and, ultimately, refers to structural modifications involving inter- and intramolecular H bond interactions, with possible consequences on protein folding. In particular, the Raman signal of amide I results from the sum of two components, one at 1640–1666 cm^-1^ which is mainly due to the β-sheet structural conformation of the proteins and one at 1675–1680 cm^-1^, linked to structural conformation with turns. A specific inversion of these two peaks was manifest in TMV samples heated at temperatures ≥85°C, showing an increase in the peak linked to protein conformation with turns (more disordered) at the expenses of the peak linked to β-sheet (Figure 4A).

These deviations in the Raman peaks linked to protein conformation are substantiated by the structural modifications of heat-treated TMV virions, for which the transition from rod to globular shape was confirmed upon treatment at 85°C (Figure 4B, upper part). Moreover, these alterations are in strong agreement with the infectivity assays performed on *N. clevelandii* plants (Figure 4B, lower part), showing a dramatic decrease in the number of local lesions on leaves inoculated with TMV particles heated at temperatures not smaller than 85°C compared to virions heated at 75°C or to untreated controls (Figure 4B, lower part and Figure 4C).

Our results confirm the suitability of RS to aid in the characterization of intact and disrupted virions previously reported by Ruokola et al. (2014), confirming the possibility to complement structural studies on virus uncoating and to evaluate alterations induced by solute-solvent ratios with dynamically spectroscopical analysis.

## Conclusion

With the Raman-DEP method here developed, profiles specific for each of the tested plant viruses were gathered, discriminating chemical differences even among viruses which are morphologically and structurally indiscernible and belong to the same taxonomic group (*Tobamovirus*). This technique allowed for the first time to build a plant virus library within a few hours, measuring purified samples directly in aqueous suspension. Moreover, this method successfully revealed spectral differences associated with structural modifications of TMV virions occurring at temperatures not smaller than 85°C, monitoring the rod-to-spherical transition of this virus, one of the most studied virus-based nanotechnological tools (Venkataraman and Hefferon, 2021).

To explore the usefulness of Raman-DEP for diagnostic purposes, future investigations with partially purified virion preparations could be conducted, aiming to simplify the purification procedures. By fine-tuning the electrical conductivity of the suspension liquids and the electric field frequency applied, selective accumulation of virions in the DEP cell and reduction of contaminating components are sought.

In sum, Raman-DEP allows a fine characterization of virions directly on purified aqueous preparations, making a turning point in the development of analytical techniques useful in virology. In this line, Raman-DEP could be adopted, for example, to test the efficacy of disinfectant or virucidal substances, allowing real-time investigation of structural and chemical alterations of virions. Finally, structural changes derived from genetic manipulation, chemical modification, or thermal treatment of virions or virion-like particles could be investigated, with important biotechnological implications (Chung et al., 2020; Venkataraman and Hefferon, 2021).

## Data availability statement

The raw data supporting the conclusions of this article will be made available by the authors, without undue reservation.

## Author contributions

AS: Data curation, Formal analysis, Investigation, Methodology, Software, Validation, Writing – original draft, Writing – review & editing. GB: Conceptualization, Formal analysis, Investigation, Writing – original draft, Writing – review & editing. SM: Formal analysis, Methodology, Writing – review & editing. AG: Formal analysis, Validation. AR: Conceptualization, Supervision, Funding acquisition, Writing – review & editing. CD’E: Formal analysis, Methodology, Writing – review & editing. MV: Formal analysis, Methodology. MC: Formal analysis, Methodology. EN: Conceptualization, Funding acquisition, Investigation, Methodology, Supervision, Validation, Writing – original draft, Writing – review & editing, Data curation. CP: Conceptualization, Funding acquisition, Project administration.

## Footnotes

^1^ https://www.european-virus-archive.com/

^2^ https://services.healthtech.dtu.dk/services/NetPhos-3.1/
