# Supplementary material for: Raman-dielectrophoresis goes viral: towards a rapid and label-free platform for plant virus characterization
Source: Front Microbiol. 2023 Nov 22;14:1292461. doi: 10.3389/fmicb.2023.1292461 (PMC10699305; doi:10.3389/fmicb.2023.1292461)
Supplement: Supplementary file 1 [file Data_Sheet_1.docx]

Raman-dielectrophoresis goes viral: towards a rapid and label-free platform for plant virus characterization

Alessio Sacco^1#*^, Giulia Barzan^1#^, Slavica Matić^2#^, Andrea M. Giovannozzi^1^, Andrea M. Rossi^1^, Chiara D’Errico^2^, Marta Vallino^2^, Marina Ciuffo^2^, Emanuela Noris^2*^, and Chiara Portesi^1^

^1^Quantum Metrology and Nano Technologies Division, National Institute of Metrological Research (INRiM), Torino, Italy

^2^Institute for Sustainable Plant Protection, National Research Council of Italy (CNR), Torino, Italy

*** Correspondence:**Corresponding Authors:

Emanuela Noris
[emanuela.noris@ipsp.cnr.it](mailto:emanuela.noris@ipsp.cnr.it)

Alessio Sacco

[a.sacco@inrim.it](mailto:a.sacco@inrim.it)

**^#^** These authors contributed equally to the work

1. **Purification of viruses**

**TMV, ToMV, and TBRFV (Tobamoviruses)**

Briefly, young *N. benthamiana* symptomatic leaves (7.5 g) collected from inoculated plants were ground in a mortar using 25 ml of phosphate buffer (PB) (pH 7.2, 0.5 M), EDTA (5 mM), Na2SO3 (0.02 M) and the mixture was filtered through Miracloth (Merck KGaA, Darmstadt, Germany). Butanol was added (8% final concentration) and the emulsion was stirred for 15 min and centrifuged at 10000× g for 30 min at 12 °C. After the addition of PEG8000 (4 % final concentration), the mixture was further stirred for 15 min in ice and then spun at 10000× g for 15 min at 4 °C in a Sorvall SS34 rotor. The resulting pellet was resuspended in PB (4ml, pH 7.2, 10 mM) and centrifuged at 10000× g for 10 min at 4 °C. Then PEG8000 and NaCl (6 % and 1M, final concentration, respectively) were added and the mixture was left in ice for 15 min. Following centrifugation at 10000× g for 15 min at 4 °C, the pelleted virions were resuspended in PB (0.5 ml, pH 7.2, 10 mM), washed twice with PB (10 mM) and spun at 10000× g for 10 min at 4 °C (Eppendorf centrifuge); the resulting supernatant containing purified virions was stored at –20 °C for further analyses.

**TSWV**

Fresh *N. benthamiana* symptomatic leaves (7 g) collected 7 days after inoculation were homogenized in a mortar using 3 volumes of extraction buffer (EB) (0.1 M Na-phosphate buffer, pH 7.0; 0.01 M Na_2_SO_3_). The homogenate was filtered through Miracloth (Merck KGaA, Darmstadt, Germany) and the filtered material was centrifuged at 12000× g for 10 min at 4 °C (Sorvall SS34 rotor). The obtained pellet was resuspended in 2 volumes of resuspension buffer (RB) (0.01 M Na-phosphate buffer, pH 7; 0.01 M Na_2_SO_3_) and the mixture stirred for 30 min at 4 °C. After centrifugation at 750× g for 15 min at 4 °C (Sorvall SS34 rotor), the supernatant was further spun at 70000× g for 15 min at 4 °C (Beckman TL100 centrifuge). The pellet containing virions was carefully resuspended in RB (0.25 ml) and layered on a sucrose density gradient (5-30 %) prepared in RB (2.5 ml/step). After centrifugation at 70000× g for 60 min at 4 °C (Beckman SW41 swinging rotor), bands were collected, diluted in 4 volumes of ddH_2_O and finally precipitated at 70000× g for 30 min at 4 °C (Beckman TL-100 centrifuge).

**CMV**

Fresh *N. tabacum* leaves (18 g) collected 11 days post inoculation were ground in a mortar with 4 volumes of cold EB (Na-citrate 0.5 M, pH 6.5; Na-thioglycolate 0.15 %). After the addition of chloroform (2 ml/g fresh tissue), the emulsion was clarified by centrifugation at 6000× g for 20 min at 4 °C (Sorvall SS-34 rotor). PEG_8000_ was added to the aqueous phase (10 % final concentration); the mixture was stirred for 15 min at 4^°^ C and then incubated in an ice bath for 1 h to precipitate virions. Samples were then centrifuged at 6000× g (Sorvall SS-34 rotor) for 20 min at 4 °C and the precipitate containing virions was resuspended in cold Na-citrate buffer (pH 7.0, 0.05 M), Triton X-100 (2 %, 0.5 ml/g of fresh tissue). After spinning at 10000× g for 5 min (Eppendorf centrifuge), the supernatant was centrifuged at 245000× g for 45 min at 4 °C (Beckman TL-100). The resulting pellet was resuspended in Na-citrate (pH 6.5, 0.05 M), layered onto a preformed sucrose gradient (10-40 %) prepared in Na-citrate (pH 6.5, 0.05 M) and spun at 25000× g for 1.5 h (Beckman, SW41 rotor). Bands were collected, diluted by 4 volumes of ddH2O and centrifuged at 70000× g for 30 min at 4 °C (Beckman TL100 centrifuge) to recover virions.

1. **Raman-Dielectrophoretic system**

The DEP cell consisted of: i) a rigid outer aluminum shell, including a carved window, allowing injection/removal of particle suspensions and the observation of their aggregation; ii) a polydimethylsiloxane (PDMS) gasket forming a sealed volume in the cell, together with a microscope cover glass; iii) an active chip containing the electrodes, wired for polarization after its connection to the sinusoidal voltage generator, forming quadrupoles and traces on a corning glass substrate.

**Table S1. List of virus isolates used in this study.**

| **Features** | **ToMV** | **TMV** | **TBRFV** | **CMV** | **TSWV N** | **TSWV G1-G1** |
| --- | --- | --- | --- | --- | --- | --- |
| Number of amino acids | 159 | 159 | 159 | 218 | 258 | 1135 |
| Molecular weight (Da) | 17745.87 | 17623.73 | 17534.68 | 24140.57 | 28900.68 | 127438.27 |
| Theoretical pI | 4.85 | 5.09 | 5.07 | 9.95 | 9.11 | 6.03 |

**Table S2. Biochemical features of the virion-associated proteins of the viruses considered.**

| **Virus species** | **Acronym** | **Family** | **Genus** | **Isolate PlaVIt** | **NCBI Acc. No.** | **EVA-GLOBAL No.** |
| --- | --- | --- | --- | --- | --- | --- |
| *Tobacco mosaic virus* | TMV | *Virgaviridae* | *Tobamovirus* | Ta9 | ON156784 | 029V-04664 |
| *Tomato mosaic virus* | ToMV | *Virgaviridae* | *Tobamovirus* | IFA9 | ON156781 | 029V-04094 |
| *Tomato brown rugose fruit virus* | TBRFV | *Virgaviridae* | *Tobamovirus* | VE492 | KT383474 | - |
| *Cucumber mosaic virus* | CMV | *Bromoviridae* | *Cucumovirus* | P132 | - | 029V-04257 |
| *Tomato spotted wilt virus* | TSWV | *Tospoviridae* | *Orthotospovirus* | P105 | DQ376178 | - |


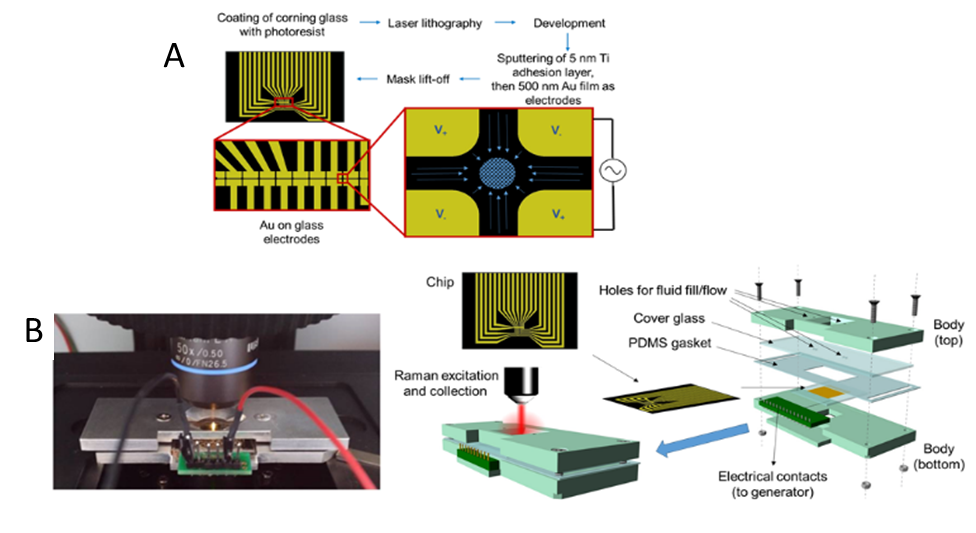


**Figure S1.** Raman-DEP in-house manufactured cell. (A) Scheme of active chip preparation. B) Image of the in-house manufactured DEP device and its positioning in the Raman instrument sample holder with the voltage connections (left) and scheme of the cell composition.

*Reprinted from Barzan, G., Sacco, A., Mandrile, L., Giovannozzi, A.M., Brown, J., Portesi, C., Alexander, M.R., Williams, P., Hardie, K.R. and Rossi, A.M., New frontiers against antibiotic resistance: A Raman-based approach for rapid detection of bacterial susceptibility and biocide-induced antibiotic cross-tolerance, Sensors and Actuators, B: Chemical, 309, 127774, Copyright (2020), with permission from Elsevier.*


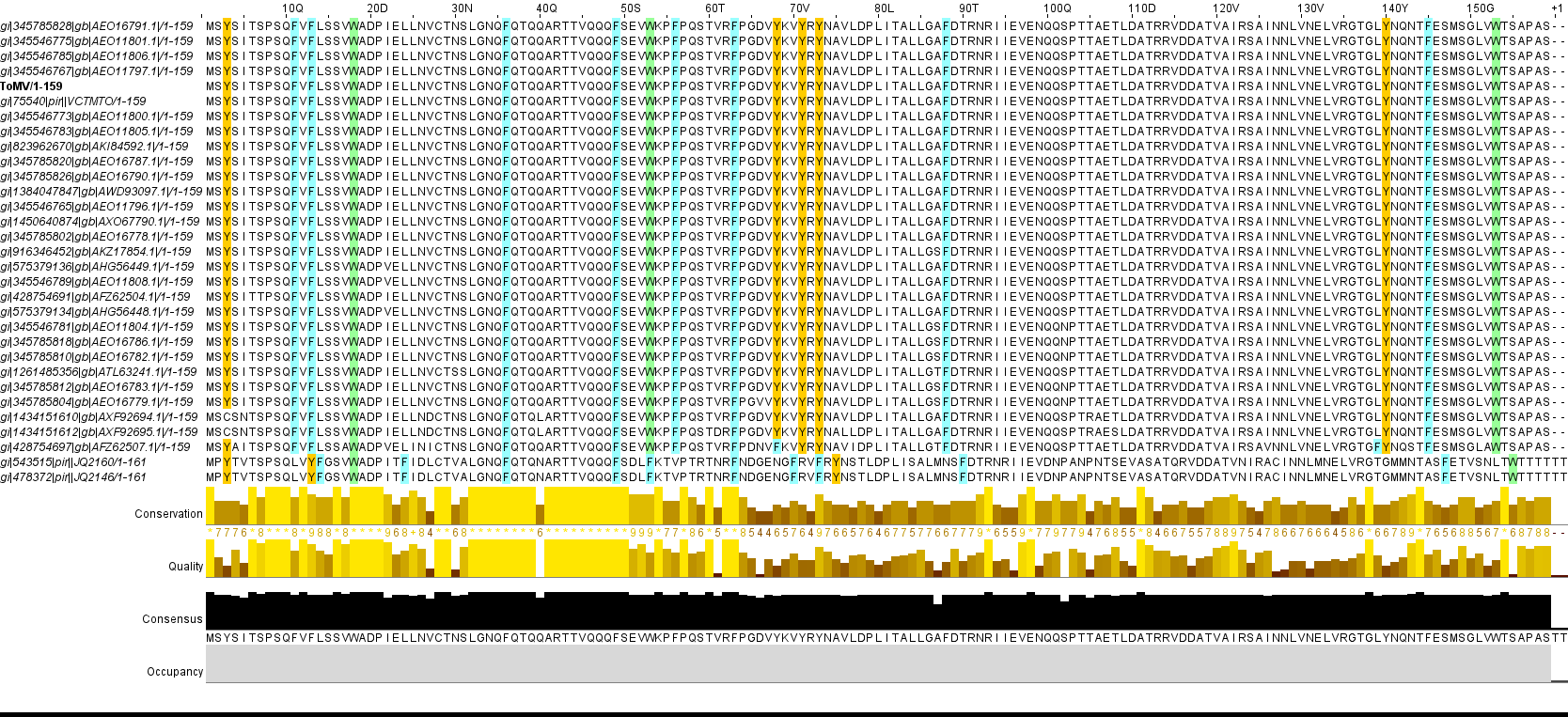

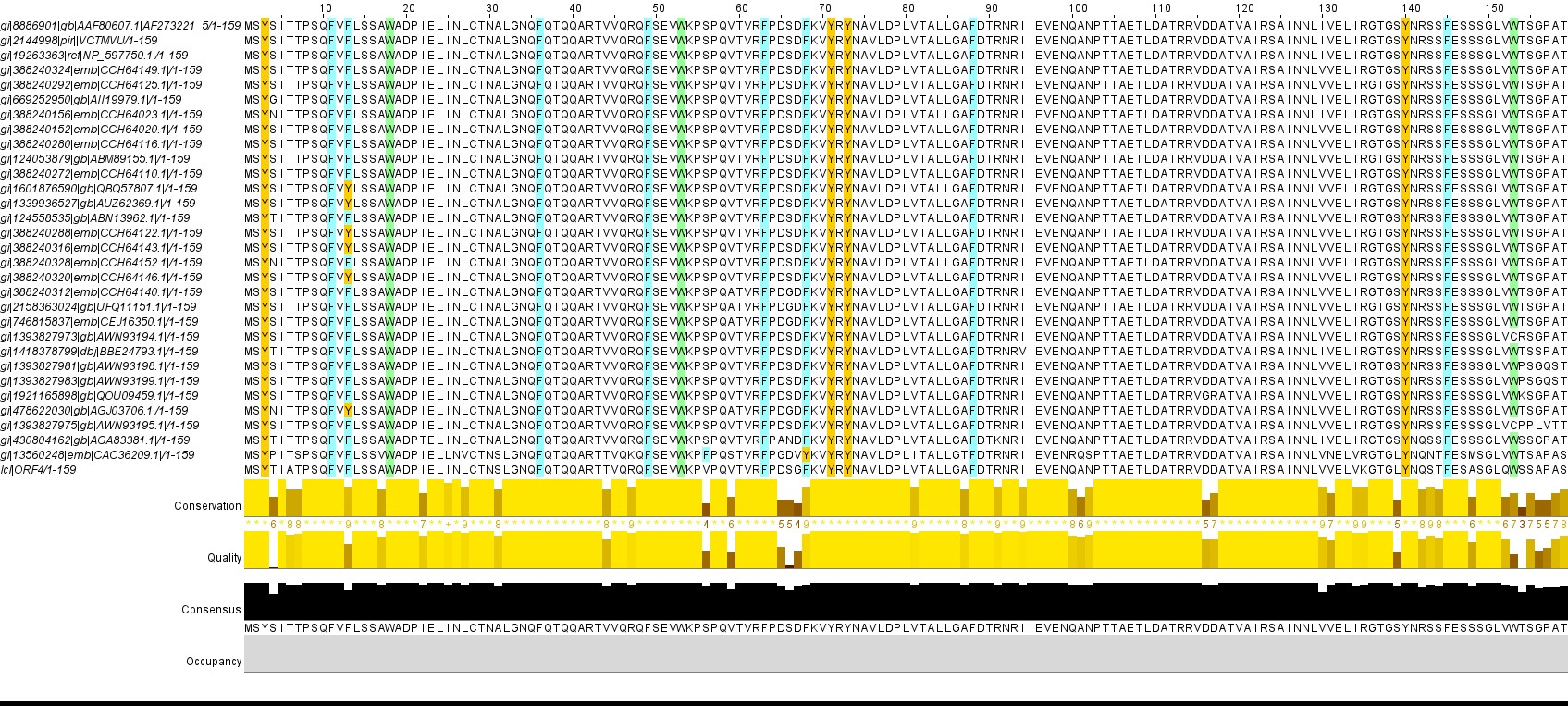


ToMV

TBRFV


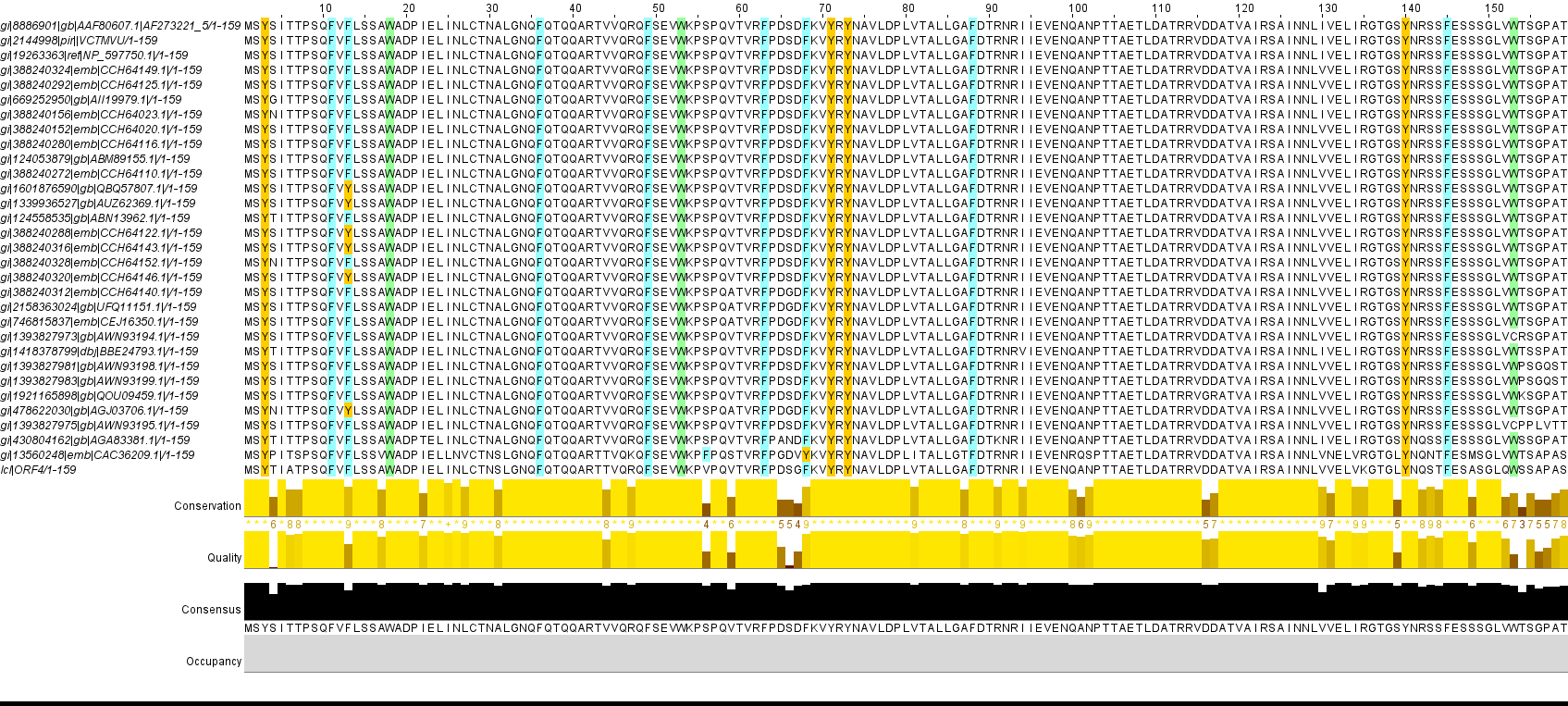


TMV

**Figure S2**. Coat protein (CP) sequence alignment of thirty isolates of TMV, ToMV, and TBRFV each, randomly selected from the NCBI Genbank database. Aromatic amino acids are highlighted in colors, as follows: tyrosine (Y) in yellow, phenylalanine (F) in cyano, and tryptophan (W) in green. The CP sequences of the isolates used in this study are shown in pink.


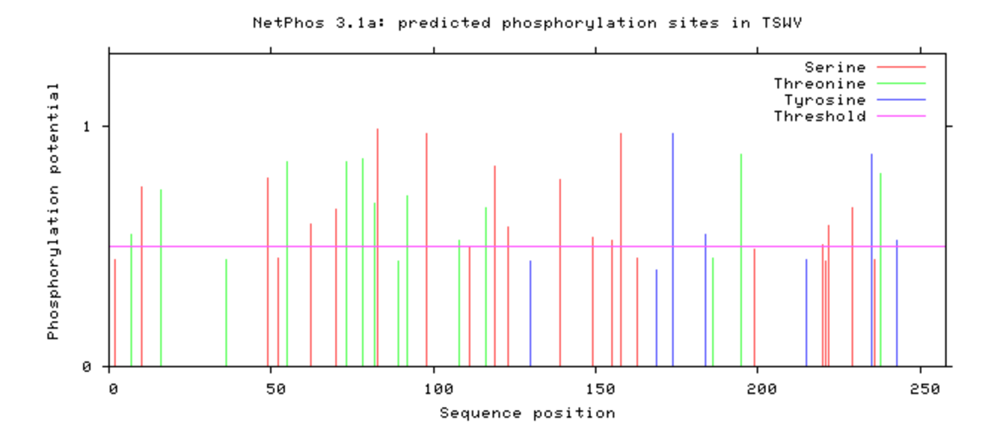
**Figure S3**. Phosphorylation site prediction of the nucleoprotein (N) sequence encoded by TSWV P105.
